# First person – Kunal Chopra

**DOI:** 10.1242/bio.042325

**Published:** 2019-02-15

## Abstract

First Person is a series of interviews with the first authors of a selection of papers published in Biology Open, helping early-career researchers promote themselves alongside their papers. Kunal Chopra is first author on ‘Zebrafish *duox* mutations provide a model for human congenital hypothyroidism’, published in BIO. Kunal is a PhD student in the lab of Enrique Amaya at the University of Manchester, investigating reactive oxygen species in wound healing and regeneration.


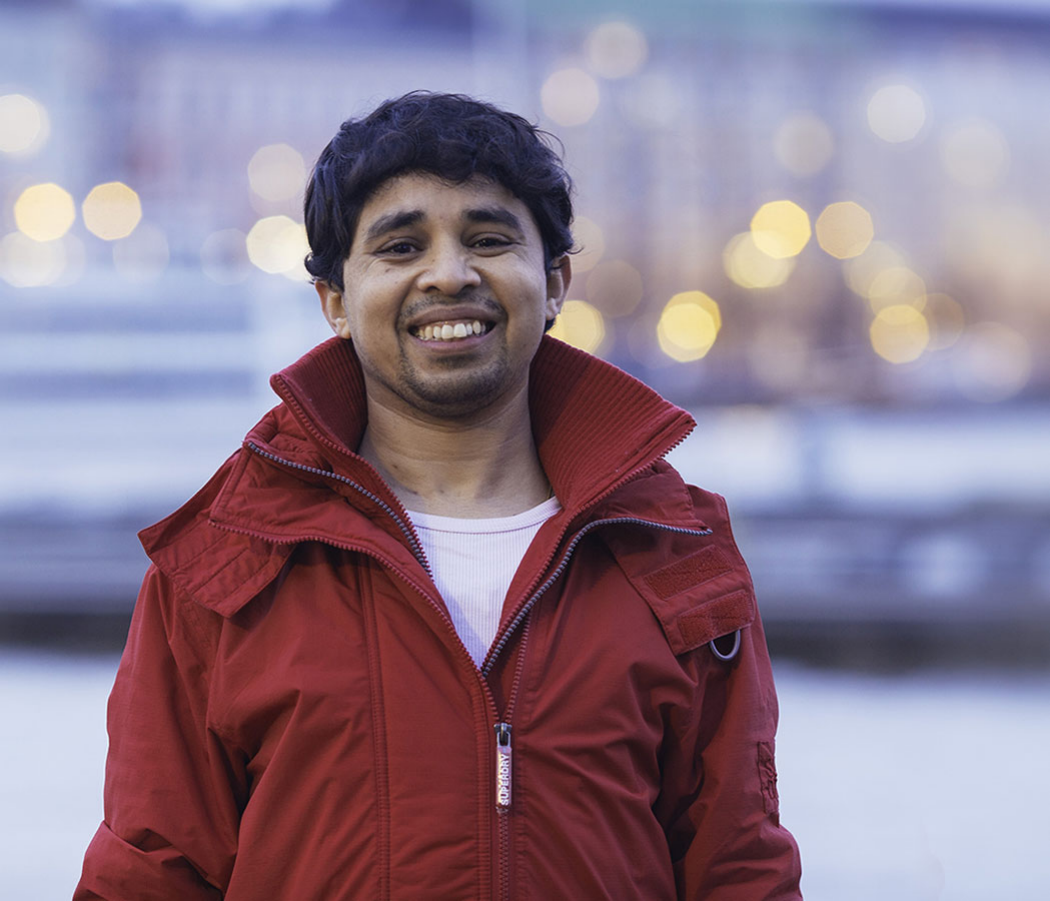


**Kunal Chopra**

**What is your scientific background and the general focus of your lab?**

I have an evergreen interest in multiple fields of biology and this is reflected in my diverse scientific background. I read zoology as an undergraduate, followed by a Master's in biological anthropology, both at the University of Delhi, India. I went on to pursue a second Master's in stem cell and regenerative medicine at the University of Sheffield, UK. I continued at Sheffield as a research assistant in the lab of Dr Henry Roehl, developing *cre* and *lox* zebrafish transgenics during which time my interest in developmental biology peaked. Presently, I am in my final year of a PhD in developmental biology, supervised by Prof. Enrique Amaya, at the University of Manchester. Our lab focuses on the molecular and cellular basis of tissue formation, repair and regeneration and we use the zebrafish and African clawed toad as model systems.

**How would you explain the main findings of your paper to non-scientific family and friends?**

We were investigating the role of a gene (*duox*), which previous research has suggested as important for tissue regeneration to occur in a scar-free manner. Therefore, we were interested in how regeneration would be affected if *duox* was mutated. Coincidentally, mutations in *duox* also lead to a common endocrine disorder, hypothyroidism, in which the individual has a deficiency of thyroid hormone. The body tries to compensate for the deficiency by enlarging the thyroid glands, a condition called goitre. In humans, this gives the neck a swollen appearance. Thyroid hormone is essential for many bodily processes including the growth and development of children as well as the ability to reproduce. Serendipitously, we found that our mutant *duox* zebrafish showed all the symptoms of hypothyroidism and we concluded that the zebrafish is a potential model for studying human hypothyroidism.

**What are the potential implications of these results for your field of research?**

Since my main focus is on regeneration, it will now be interesting to determine the role of thyroid hormone on regeneration, in addition to *duox*. Importantly, it will be necessary to dissect out the individual roles of thyroid hormone and *duox*, to ensure that neither is a confounding factor on observations.

**What has surprised you the most while conducting your research?**

My model organism, the zebrafish, never ceases to surprise. That we found a host of phenotypes in an animal that is just 5 cm long is a testament of how much potential it holds. Further, genetic conservation has always fascinated me! Outwardly, humans and fish are like chalk and cheese but to see the evolutionarily conserved roles of genes reconcile them (and other groups of animals) is extremely exciting.

“Outwardly, humans and fish are like chalk and cheese but to see the evolutionarily conserved roles of genes reconcile them…is extremely exciting.”

**Figure:**
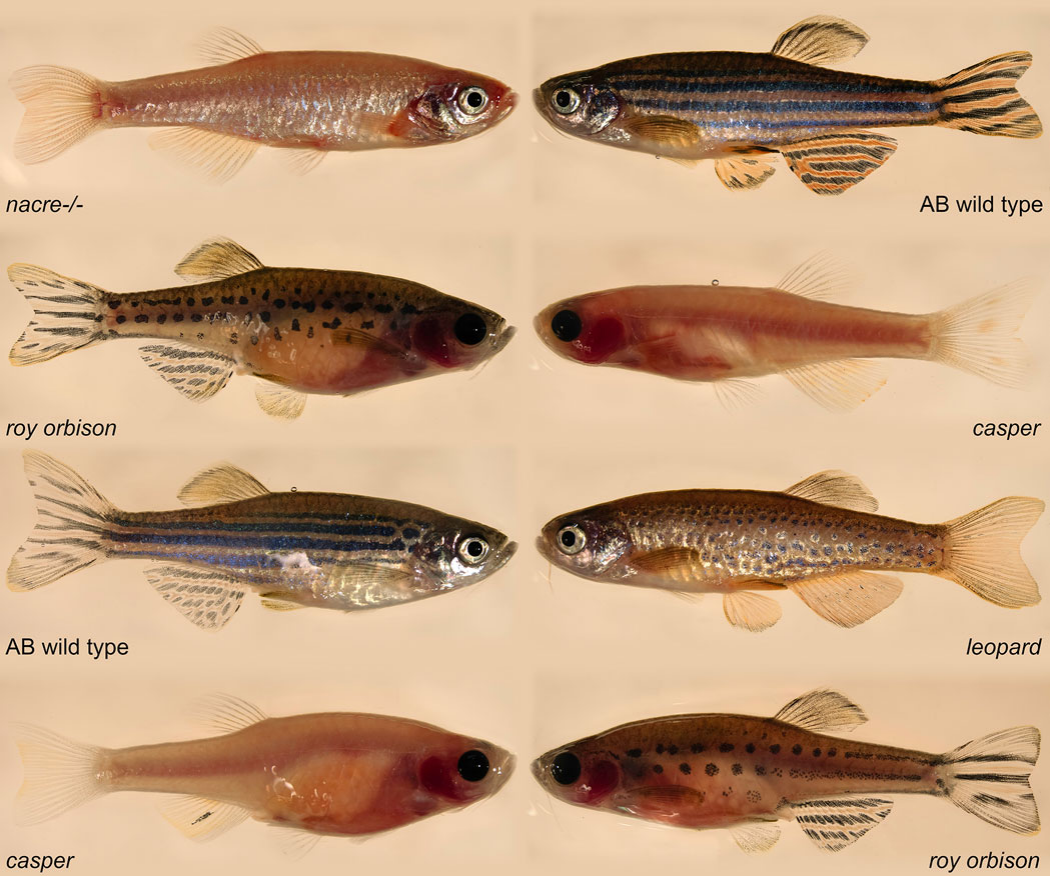
**Zebrafish strains.**

**What, in your opinion, are some of the greatest achievements in your field and how has this influenced your research?**

The establishment of the zebrafish as a versatile animal model has been an enormous achievement. In tandem, the zebrafish mutation project (ZMP) (Kettleborough et al., 2013) has provided an extensive spread of knockouts (commonly referred to as Sanger mutants) for modeling disease. Equally, the refinement of CRISPR-mediated mutagenesis (Moreno-Mateos et al., 2015) has given us a very efficient method for generating targeted mutations. For my research in general, I use CRISPR and Sanger mutants. For this work in particular, I chose to use two alleles of *duox* from the ZMP to check for concordance and make our findings more robust. Overall, a robust model coupled with extensive resources and techniques have ensured that this will be my model organism of choice for future research endeavours.

**What changes do you think could improve the professional lives of early-career scientists?**

Up until a few years ago, PhD students would have found themselves solely invested in their research, which is rewarding if the project consistently yields results, but taxing if otherwise. Nowadays, with entrepreneurship and knowledge transfer opportunities, early-career researchers can break the monotony of research and also determine for themselves if they want to branch out. Research faculties at Universities, in the UK at least, are also doing more in terms of ensuring student engagement by way of organising extra-curricular and social activities. Finally, students themselves must try to regularly engage in an activity of their choice because it helps one to remain sane and motivated.

**What's next for you?**

Instinctively, I feel I will be heading on to a post-doctoral position after the PhD. A project that involves cross-species comparisons would be especially tempting. Bench research feels like my absolute calling. However, in light of the present political flux and the uncertainty that it has cast on research funding, I wish to remain flexible about plans for the long-term future.
